# PRP Therapy for Stress Urinary Incontinence and Pelvic Organ Prolapse: A New Frontier in Personalized Treatment?

**DOI:** 10.3390/jpm15060214

**Published:** 2025-05-22

**Authors:** Anna Pitsillidi, Laura Vona, Stefano Bettocchi, Sven Schiermeier, Günter Karl Noé

**Affiliations:** 1 Department of OB/GYN, Rheinland Klinikum Dormagen, Dr.-Geldmacher-Straße 20, 41540 Dormagen, Germany; guenter.noe@uni-wh.de; 2 Department of OB/GYN, University of Witten Herdecke, Alfred-Herrhausen-Straße 50, 58455 Witten, Germany; sven.schiermeier@uni-wh.de; 3 Department of Medical and Surgical Sciences, Institute of Obstetrics and Gynaecology, University of Foggia, Via Antonio Gramsci, 89, 71122 Foggia, Italy; laura.vona@unifg.it (L.V.); info@stefanobettocchi.com (S.B.)

**Keywords:** prolapse, stress urinary incontinence, platelet-rich plasma, PRP injections

## Abstract

**Background:** Pelvic organ prolapse (POP) and stress incontinence (SUI) are very common medical conditions, affecting women’s quality of life worldwide. Current surgical and conservative therapies often yield variable outcomes and carry risks of complications or recurrence. Platelet-rich plasma (PRP) has emerged as a promising regenerative approach in various medical disciplines. Its application in urogynecology remains relatively new and emerging. The objective of this study was to review and consolidate existing evidence regarding the application of PRP injections for treating POP and/or SUI. **Methods:** This scoping review was conducted in accordance with the Preferred Reporting Items for Scoping Reviews (PRISMA-ScR). The search strategy included MEDLINE (PubMed), Web of Science, and Scopus databases, covering articles published up to February 2025, with no restrictions on publication date. **Results:** We included in our review a total of 13 manuscripts and 320 patients at the end of the screening process. A total of ten SUI studies, comprising 273 patients, and three POP studies, involving 47 patients, satisfied all the review criteria. Both clinical entities reported high subjective improvement following PRP treatment. Moreover, PRP appeared to have no significant adverse effects. **Conclusions:** Our scoping review suggests that PRP may have potential benefits in the treatment of POP and SUI. Nevertheless, the current evidence on its application in this area remains limited. Therefore, well-designed, large-scale randomized controlled trials (RCTs) with extended follow-up periods are urgently needed, in the era of personalized medicine.

## 1. Introduction

Pelvic organ prolapse (POP) is a very common medical condition, affecting women’s quality of life worldwide [1,2]. Approximately 19% of women will require surgery for POP at least once in their lifetime. Numerous techniques haven been employed for POP treatment [3]. Unfortunately, relapse is common, with 29% undergoing at least one additional procedure [4] and recurrence of anterior compartment prolapse being the most common [5]. In recent years, vaginal meshes have been widely utilized; however, due to their high complication rates, the Food and Drug Administration (FDA) issued reports informing both patients and physicians about the associated risks [6].

Stress urinary incontinence (SUI) is also a prevalent condition that significantly impacts women’s daily lives [7]. According to the International Continence Society (ICS), SUI is the unintentional loss of urine triggered by activities that elevate abdominal pressure, such as coughing, sneezing, or physical exertion, and it appears to be the most common type of urinary incontinence (UI) [8,9]. The therapeutic options for SUI are both conservative and surgical. If conservative treatments (lifestyle adjustments, pelvic floor muscle training-PFMT-, pessaries, and pharmacological treatment) are ineffective, surgical options for managing incontinence include a range of procedures such as synthetic mid-urethral slings, colposuspension, autologous fascial slings, and urethral bulking agents [10,11,12]. Nevertheless, surgical treatment for SUI carries potential risks and complications, while bulking agents can be costly and may not be accessible in many countries [13,14].

Due to the high prevalence of these clinical entities and their strong impact on women’s quality of life, multiple innovative alternative methods have been suggested to enhance the use of minimally invasive therapies lately. The most popular one is platelet-rich plasma (PRP). The term PRP was initially introduced in the 1970s to refer to plasma that contained a higher concentration of platelets compared to peripheral blood. In 1974, Kohler and Lipton, while researching fibroblast physiology, discovered that platelets could play a crucial role as growth stimulants [15]. PRP is shown to be rich in growth factors and able to promote angiogenesis, neuroprotection, neural regeneration, inflammation regulation, and wound healing, all of which contribute to improved organ function [16,17]. In the market, there are many different kits, such as the RegenKit^®^ A-PRP, or TruPRP^®^, available for the preparation of a PRP solution with varying platelet concentrations and additional ingredient compositions [18]. PRP seems to be a safe therapeutic option due to its autologous nature which eliminates the risk of immune reactions and the potential transmission of microorganisms from external donors [19]. Moreover, it can be cost-effective due to its quick and straightforward preparation, requiring minimal costs [20]. However, PRP is contraindicated for certain patient groups, including those with coagulation disorders, cancer, pregnant women, or individuals with infectious diseases [21]. Although PRP has been extensively utilized across various medical specialties, including orthopedics, urology, ophthalmology, aesthetic medicine, and dermatology, its application in urogynecology remains relatively new and emerging. The objective of this study was to review and consolidate existing evidence regarding the application of PRP injections for treating POP and/or SUI.

## 2. Materials and Methods

### 2.1. Search Strategy

**Figure 1:**
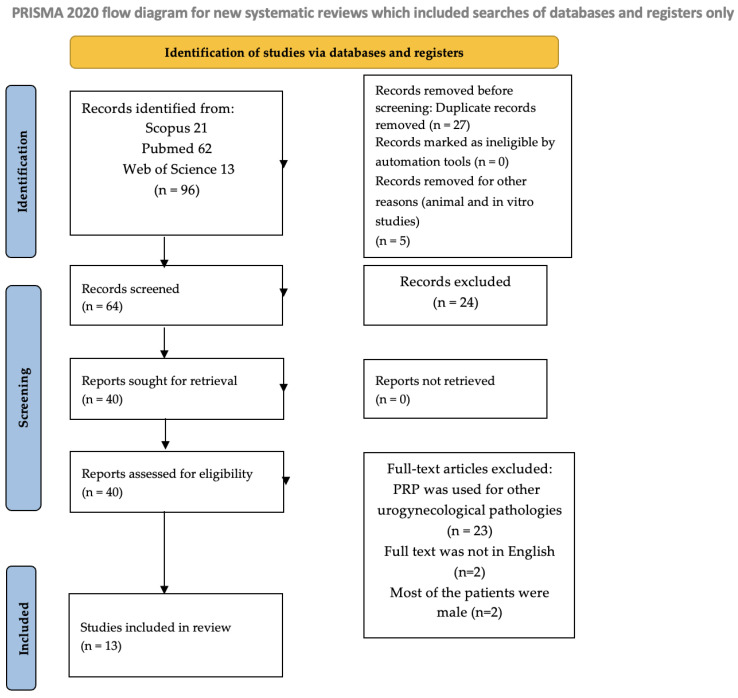
Flow diagram.

This scoping review was performed following the Preferred Reporting Items for Systematic reviews and Meta-Analyses extension for Scoping Reviews (Supplementary Table S1: PRISMA-ScR) guidelines [22]. Two researchers (L.V. and A.P.) conducted a comprehensive search of the Web of Science, Scopus, and MEDLINE (PubMed) databases, including studies published up to February 2025, without applying any historical restrictions. The search strategy involved various combinations of the following MeSH terms: Platelet-Rich Plasma, Stress Urinary Incontinence, Pelvic Organ Prolapse, Urogynaecology. For the articles’ selection, we included articles that focused on PRP treatment for UI and/or POP. The authors also searched for literature on PRP treatment of urgency alone, but by the target date, no articles were found. The PRISMA flow diagram of the selection process is provided in Figure 1.

### 2.2. Eligibility Criteria

The following study designs were deemed eligible for inclusion: case reports, randomized controlled trials, prospective controlled studies, prospective cohort studies, retrospective studies, and case series. Only full-text articles published in English were considered. In contrast, systematic reviews, meta-analyses, letters to the editor, and conference abstracts were excluded from the analysis. However, the reference lists of relevant reviews were screened to identify any additional eligible studies. Research with unclear, incomplete, poor-quality data, or outcomes that could not be quantified was also excluded. Articles whose full text was not written in English as well as studies in which patients were treated for other gynaecological and urogynaecological conditions were excluded. Furthermore, two case series were excluded due to a predominantly male patient population and the lack of clearly differentiated demographic data and outcomes between male and female participants. Therefore, the data quality was poor [23,24]. In vitro and animal studies were excluded [25,26,27,28].

### 2.3. Data Acquisition and Risk of Bias

All records identified through database searches were screened for publication year, citation, title, authors, abstract, and full text. Duplicate entries were manually detected and removed by two independent reviewers (L.V. and A.P.). During the eligibility assessment, the same two reviewers independently screened the titles and abstracts of the remaining records, excluding those not relevant to the research question. Full texts of potentially eligible studies were then independently assessed for inclusion. Any discrepancies between the reviewers were resolved through discussion and consensus. The methodological quality of the included studies was appraised using the JBI Critical Appraisal Checklists for Case Reports and Clinical Trials (Supplementary Table S2).

### 2.4. Data Synthesis and Statistical Analysis

We included in our review a total of 13 manuscripts and 320 patients at the end of the screening process (Supplementary Tables S3 and S4 (demographic table) Tables S1 and S2 (data table)). Ten studies investigating SUI (4 Randomized Control Clinical Trial and 6 Prospective studies) and 273 patients and three studies investigating POP (1 Randomized Control Clinical Trial and 2 Prospective studies) and 47 patients meet all the review requirements. We examined in our review the patients’ demography, their obstetrical history, inclusion and exclusion criteria, the PRP kit used, injection protocol, considered scores, results, and follow-up time from the baseline (first PRP injection). Heterogeneity of study type (RCT and observational study) and the high number of different outcomes considered prohibited systematic analysis; thus, a scoping review was performed. However, demographic and participant characteristics were comparable. A mean was used to summarize continuous variables and a percentile was used for dichotomous variables. Because it is a Scopus review and not a systematic review, this review was not registered.

## 3. Results

### 3.1. Pelvic Organ Prolapse

#### 3.1.1. Demographics and Participant Characteristics

The mean age of the patients was 57.3 years, with a mean Body Mass Index (BMI) of 25.8 kg/m^2^ and a mean parity of 2.9. Seventy-four percent of the patients were post-menopausal. In the study by Atilgan et al., patients who underwent cystocele repair for other prolapse compartments or concomitant surgery for SUI were excluded [29]. In contrast, the studies by Einarsson et al. and Gorlero et al. included patients who underwent concomitant prolapse or SUI surgery, with 77.8% of the patients in the former and 100% in the latter receiving such procedures [30,31].

Gorlero et al. reported that 60% of the patients had a Pelvic Organ Prolapse Quantification (POP-Q) stage II prolapse, while 40% had a stage II prolapse [31]. Atilgan et al. reported Aa and Ba points of +3 and +3.6, respectively [29].

In the studies by Einarsson et al. and Atilgan et al., a history of prolapse surgery was an exclusion criterion [29,30]. Conversely, Gorlero et al. included patients with a history of prolapse surgery and those at high risk of recurrence [31].

Regarding platelet preparation, the specific kit used in the study by Einarsson to prepare autologous platelet gel (APG) was not specified [30]. Gorlero et al. employed a Vivostat system to prepare platelet-rich fibrin (PRF), which was then sprayed directly onto the surgical site [31]. Atilgan et al. utilized a Vacutainer Kit to PRP [29]. In all studies, platelet preparation was applied during surgery, and cystocele or other prolapse repairs followed standard surgical techniques. No adverse events were observed during follow-up in 100% of the patients. The mean follow-up duration across studies was 37.5 months.

#### 3.1.2. Scores and Results

In Atilgan et al.’s RCT-single blind, 28 cases were treated with PRP injection into the pubocervical fascia and colporrhaphy and 28 controls only with colporrhaphy. The Aa and Ba points’ means were significantly lower in cases than in controls at 48 months follow-up (*p* = 0.001 and *p* = 0.002, respectively). Symptomatic (evaluated by Pelvic Floor Distress Inventory-PFDI), anatomic recurrence (POP-Q > 1), and reoperation rate were significantly lower in cases than in controls (*p* = 0.008, *p* = 0.001 and *p* = 0.001, respectively). Only 3.8% of the patients in the cases’ group had a symptomatic anatomic cystocele recurrence and were reoperated. Subjective success (evaluated by Patient’s Global Impression of Improvement-PGI-I) was significantly higher in cases, with a rate of 89% (*p* = 0.012) [29].

In their study involving a cohort of nine patients, Einarsson et al. collected punch biopsy specimens both before surgery and during follow-up to assess tissue weight and collagen content. No statistically significant difference in collagen content was observed at follow-up (*p* = 0.63); however, tissue samples collected during surgery were significantly heavier than those obtained at follow-up (*p* = 0.004). The POP-Q assessment demonstrated statistically significant improvements at 3 months postoperatively at points Aa and Ba. At the 20-month follow-up, only point Aa showed a statistically significant difference compared to baseline. In patients who completed the follow-up (77.8%), at 20 months, the subjective failure rate was 12.5% and the objective failure rate was 66.7%. The reoperation rate was 12.5% for a recurrent and symptomatic stage II cystocele and enterocele [30].

Furthermore, Gorlero et al. conducted a prospective observational study enrolling ten patients. The overall efficacy rate in terms of anatomical reconstruction was 80% for POP-Q stage 0 and 20% for Pop-Q stage I based on the Prolapse Quality of Life (P-QoL) questionnaire; repair of the vaginal wall descent led to a 20% increase in the number of patients engaging in sexual activity, with no cases of dyspareunia reported postoperatively. Urinary and bowel symptoms showed complete resolution by 24 months. At follow-up, all patients presented with normal scarring, as assessed by the Vancouver Scar Scale (Table 1) [31]. 

### 3.2. Stress Urinary Incontinence

#### 3.2.1. Demographics and Participants Characteristics

In the included clinical trials, both groups (case and control) had similar demographics and participant characteristics. For our statistics, we considered only the case group.

The mean age was 53.1 years. BMI was reported in seven studies and the mean was 28.4 kg/m^2^ [32,33,34,35,36,37,38]. Behnia-Willson et al. reported only the percentage of obese patients [39]. Saraluck et al. excluded obese patients from their study.

The mean parity was reported in six studies and was 2.5 [32,33,35,36,38,40]. Two studies reported only that the patients had at least one vaginal delivery [34,39]. Menopausal status was reported in six studies, with 47.3% of patients across these studies being post-menopausal [32,34,35,36,38,39]. Five studies documented the mean VAS pain score during PRP injection, with an average of 2.6 [32,33,34,35,37].

In three studies, prior surgery for POP was listed as an exclusion criterion [32,39,40]. Specifically, Grigoriadis et al. explicitly stated that hysterectomy for POP was an exclusion criterion [32]. In contrast, Saraluck et al. and Ashton et al. reported the percentage of patients with a history of hysterectomy in their study populations [34,37]. Current POP was an exclusion criterion in seven studies [32,33,34,36,39,40,41]. In the study by Chiang et al., a prior suburethral sling procedure was an inclusion criterion [41]; however, previous surgery SUI was an exclusion criterion in five studies [32,33,34,36,40]. Additionally, detrusor overactivity or urgency symptoms identified on urodynamic testing were exclusion criteria in four studies [32,33,34,40].

Regarding the kit used for PRP preparation, four studies utilized the RegenKit system [33,34,35,39]. Ashton et al. employed the Arthrex Autologous Conditioned Plasma Double Syringe System [37], Grigoriadis et al. used the OMNIPLEX Gyno kit [32], and Tahoon et al. utilized the Golden VAC kit [38]. In contrast, three studies did not specify the PRP kit used, providing only a description of the preparation protocol [36,40,41].

Behnia-Willson et al. administered a combination therapy, employing the MonaLisa Touch SmartXide2 V2LR laser as an adjuvant to PRP injection [39].

Regarding the injection protocol, in three studies, patients received a single PRP injection [35,37,38]. In another three studies, two injections were administered per patient [32,33,34]. Ural et al. and Behnia-Willison et al. applied three PRP injections [36,39], while Chiang et al. administered four [41]. Furthermore, in the study conducted by Daneshpajooh et al., following the initial treatment, some patients chose to receive a second and, in some cases, a third injection [40]. In all of these studies, subsequent injections were administered within a 4–6-week interval after the previous treatment.

The mean follow-up duration was 8 months. Adverse events were reported in nine studies; however, no severe adverse events were documented following their completion.

#### 3.2.2. Scores and Results

In the double-blind, RCT by Grigoriadis et al., two groups were evaluated. The case group was treated with PRP, while the control group received a sham treatment (0.9% sodium chloride). Significant improvements were observed in the case group at both 3 and 6 months in terms of the Incontinence Questionnaire—Female Lower Urinary Tract Symptoms (ICIQ-FLUTS), incontinence, total ICIQ-FLUTS mean score, PGI-I, and the 1 h pad test, whereas no such improvements were noted in the control group (*p* < 0.001). At the 6-month follow-up, the subjective cure rate was significantly higher in the PRP group (32%) compared to the sham group (4%) (*p* < 0.01). However, no patients were objectively cured during the 6-month follow-up [32].

In the single-blind, RCT conducted by Ashton et al., two groups were evaluated. The case group was treated with PRP, while the control group received a sham treatment (0.9% sodium chloride). No statistically significant differences were observed between the two groups in the primary outcomes, including PGI-I improvement and provocative cough stress test (CST). Additionally, there were no significant differences in Female Sexual Function Index (FSFI), Incontinence Quality of Life (I-QoL), and Questionnaire for Urinary Incontinence Diagnosis (QUID) scores at any follow-up interval [37].

In the single-blind RCT performed by Saraluck et al., the case group received treatment with PRP in conjunction with pelvic floor muscle training (PFMT), while the control group was treated only with PFMT. After a 5-month follow-up, a statistically significant improvement in the 1 h pad test was observed between the two groups (*p* < 0.05). Furthermore, the case group demonstrated significantly greater improvements in I-QoL scores, as well as in items 11a and 11b of the ICIQ-FLUTS, compared to the control group (*p* < 0.001). PGI-I scores also showed a significant advantage in the case group (*p* < 0.05). Additionally, 90% of patients in the case group reported a subjective improvement of at least 50% in their symptoms of SUI [34].

In the prospective RCT by Daneshpajooh et al., the case group received PRP treatment, while the control group underwent mid-urethral sling surgery. At the 1-month follow-up, the CST yielded negative results for 70% of patients in the PRP group, compared to 80% in the control group. However, no statistically significant differences were observed between the groups at both the 1-month and 3-month follow-ups. Results of the ICIQ, I-QoL, and Urinary Distress Inventory (UDI-6) were significantly improved before and after treatment in both groups. Nevertheless, the response to the mid-urethral sling procedure was superior, with a statistically significant difference between the two groups [40].

In the prospective observational pilot study conducted by Athanasiou et al., significant reductions were observed in the 11a question score, filling score, incontinence score, and total mean score of the ICIQ-FLUTS questionnaire (*p* < 0.001). At the 6-month follow-up, 10% of patients reported feeling subjectively cured, while 80% of patients experienced subjective improvement. The PGI-I demonstrated significant improvement in 80% of the patients throughout the follow-up period. A mean reduction of 50.2% in urine loss was observed in the 1 h pad test at the 6-month follow-up, with 10% of participants considered objectively cured [33].

In the study by Chiang et al., the patients were treated with four PRP injections. Following the PRP treatment, 80.8% of patients reported a positive response in alleviating SUI, with 50% achieving a GRA ≥ 2, which was considered a successful outcome. At 3 months, 46.2% of patients achieved complete continence and were pad-free (dry rate), while 26.9% maintained this outcome at 15 months. Additionally, 53.8% of patients remained mildly incontinent but with an acceptable outcome. The VAS score for SUI showed significant improvement immediately after the first PRP injection, with further consolidation following subsequent injections. This therapeutic effect was sustained throughout the 1-year follow-up period (*p* < 0.001). At 3 months, both UDI-6 and Incontinence Impact Questionnaire (IIQ-7) scores showed significant reductions (*p* < 0.001). However, no significant differences were observed in the videourodynamic study VUDS) parameters between baseline and post-treatment, with the exception of abdominal leak point pressure (ALPP) [41].

The prospective observational pilot study conducted by Behnia-Willson et al. assessed the Australian Pelvic Floor Questionnaire (APFQ), specifically question 6. At baseline, 0% of patients reported occasional or no stress urinary symptoms, while 100% reported frequent or daily symptoms. At the 3-month and 12–24-month follow-ups, 66.2% and 62.1% of patients, respectively, reported occasional or no symptoms. All symptoms assessed by the APFQ showed significant improvement from baseline to the end of follow-up [39].

In the observational study by Long et al., significant improvements in incontinence were observed at both 1 month and 6 months post-treatment, as measured by the Incontinence Questionnaire-Short Form (ICIQ-SF), UDI-6, and IIQ-7 in the 20 patients enrolled. Urodynamic studies revealed a significant increase in both residual urine and bladder volume at the first sensation to void [35].

In the observational study conducted by Ural et al., on the ICIQ-SF questionnaire, the rate of clinical improvement was 79.4% at 1 month and 64.7% at 6 months post-treatment. Scores on the ICIQ-SF, UDI-6, IIQ-7, and POPDI-6 at both 1 and 6 months demonstrated statistically significant improvement compared to baseline (*p* < 0.001) [36].

Tahoon et al. observed a significant increase in residual urine volume and bladder volume at the first sensation to void. Following PRP treatment, the average 1 h pad test result was 2.55 g, representing a reduction of 2.8 g from baseline. Significant improvements in incontinence were observed at both 1- and 3-months post-treatment, as measured by the ICIQ-SF, UDI-6, IIQ-7, OABSS, and FSFI. According to the ICIQ-SF, 54% of patients experienced symptom improvement or cure (Table 2) [38].

## 4. Discussion

Our scoping review suggests that PRP therapy may lead to symptomatic improvement in POP and female SUI. However, it is important to emphasize that, due to significant clinical heterogeneity, definitive conclusions regarding the efficacy of PRP cannot yet be established.

POP has been linked to a marked reduction in collagen content within the pelvic connective tissues when compared to healthy individuals. This observation has provided the rationale for exploring PRP as a therapeutic option, given its known ability to enhance collagen synthesis and facilitate tissue repair through the release of growth factors involved in wound healing and extracellular matrix remodeling [42,43]. Despite its theoretical benefits, the current body of evidence is limited to small-scale studies and case series, with considerable heterogeneity in methodology, patient selection, and outcome measures. In all included studies, PRP was administered via injection into the pubocervical fascia, with findings indicating higher anatomical success rates and lower reoperation rates compared to control groups [29,30,31]. Due to the high heterogeneity regarding the validation questionnaires or scales used, it seems rather problematic to make any concrete conclusions about the impact of PRP in patients’ quality of life. However, a subjective success rate of 89%, as measured by the PGI-I questionnaire, was reported [29], while the VAS scale indicated a subjective failure rate of 12.5% in a pilot study by Einarsson et al. [30]. Furthermore, a 20% increase in sexual activity evaluated by P-QoL was demonstrated by Gorlero et al. [31].

Regarding SUI, all studies included in our scoping review utilized peri-urethral PRP injections, targeting the anatomical deficits of the urethra. This approach was based on PRP’s regenerative potential, including its capacity to enhance tissue repair, promote angiogenesis, and provide neuroprotective effects [44]. As observed in the POP trials, the use of heterogeneous questionnaires in the SUI studies similarly complicates outcome evaluation and restricts the ability to draw definitive conclusions. Nevertheless, each study reported a statistically significant improvement based on the specific questionnaire utilized. Daneshpajoch et al., however, demonstrated significantly greater improvement in outcomes with the mid-urethral sling procedure [40]. Regarding subjective cure rates, Grigoriadis et al. reported a significantly higher rate in the PRP group (32%) compared to the control group (4%) (*p* < 0.01) [32]. Comparable findings were reported by Saraluck et al., with 90% of patients in the intervention group experiencing a subjective improvement of at least 50% in their SUI symptoms [34]. Athanasiou et al. reported a 10% subjective cure rate, while 80% of patients experienced a perceived improvement in their symptoms [33].

PRP injections have been widely studied across diverse medical fields—including wound care, orthopedics, urology, dental surgery, and cosmetic procedures—and have consistently demonstrated a favorable safety profile, with no reports of serious adverse events such as infections, bleeding, or nerve damage [45,46]. Consistent with these findings, our scoping review revealed that none of the patients receiving PRP injections experienced serious complications. Only a small proportion—less than 1% (3 out of 317 patients)—reported minor adverse events, including vaginal spotting, discomfort, and urinary straining. These issues were self-limiting and resolved following a brief period of intermittent self-catheterization [37,41].

It is noteworthy that there are limitations that need to be addressed. First of all, as already mentioned, the considerable variability across the included studies—in PRP preparation methods, dosage, injection sites, and treatment duration—makes a systematic review unfeasible. Furthermore, the small number of enrolled patients and the short follow-up limit the generalizability of the results. Finally, it should be acknowledged that not all studies reported adverse effects following the application of PRP, representing a noteworthy limitation in the evidence base of the included trials.

## 5. Conclusions

According to our scoping review, PRP may contribute to the treatment of POP and SUI. However, evidence on PRP utilization in this field is scarce, and there is still no consensus about the ideal number or frequency of treatment sessions. Thus, the need for larger-scale RCTs with longer follow-up is of great importance.

## Figures and Tables

**Table 1:** Characteristics and data extracted of included studies about PRP in POP treatment.

Author	Study Type and Patients Number	Protocol	Scores	Results	Follow-Up from the Baseline (First Injection) and Adverse Events
Einarsson J.I. et al. [30]	Prospective cohort study n = 9	A 6 mm punch biopsy was obtained from the anterior vaginal wall, approximately 4 cm posterior to the urethra and 2 cm lateral to the midline, at a single vaginal site. The cystocele repair followed standard surgical principles. The APG was applied after plication of the pubocervical fascia and before closure of the vaginal epithelium. Three months postoperatively, the patients returned for a follow-up examination and a second 6 mm punch biopsy.	**POP-Q** before surgery, 3 months after surgery, and at a mean of 20 months after surgery. Objective recurrence of anterior compartment prolapse was defined as **point Aa** at a position of −1 or greater. Subjective recurrence was defined as patient satisfaction less than 3 on a 5-point **VAS**. **Collagen content** was measured and compared.	**VAS**: Patient satisfaction mean at 3 months was 4.1 and at 20 months was 4.3. In patients who completed the follow-up (77.8%), at 20 months, the **subjective failure rate** was 12.5% and the **objective failure rate** was 66.7%. The **reoperation rate** was 12.5% for a recurrent and symptomatic stage II cystocele and enterocele. **POP-Q** showed statistical significance at 3 months at points Aa and Ba, but at 20 months follow-up, only a statistically significant difference at point Aa as compared with baseline was found. No significant differences existed between **collagen content** at 3 months (*p* = 0.63). The samples submitted during surgery were significantly heavier than samples taken during the follow-up (*p* = 0.004).	3 and at a mean of 20 months
Gorlero F. et al. [31]	Prospective observational study n = 10	Anterior repair was performed for transverse and midline defects plus application of PRF. Posterior repair was performed plus PRF after any posterior defects were identified. Perineorrhaphy was included when necessary. Apical repair was carried out using PRF in combination with reattachment of the vaginal cuff and the muscularis layer of the anterior vaginal wall to the uterosacral ligaments. Enterocele repair was performed concurrently with PRF application. In patients with occult stress urinary incontinence, plication of the endopelvic fascia at the mid-urethral level was performed in conjunction with PRF.	Italian version of the **P-QoL (Version 4) Questionnaire**, Scar quality and wound healing were assessed with the **Vancouver Scar Scale.** Prolapse repair was considered to be anatomically successful if the patient was asymptomatic and classified as stage 0 according to the **POP-Q system.**	**POP-Q**: The overall **efficacy** rate was 80% for stage 0 and 20% for stage I. **P-QoL**: when vaginal wall descent was repaired, the patients who had **sexual activity** increased by 20%, and no women had dyspareunia after surgery. **Urinary and bowel symptoms** had improved by 100% at 24 months. At follow-up, all patients exhibited normal scar formation (Vancouver Scar Scale). No keloid formation was observed. No patients reported pruritus or any type of pain in the surgical areas. There were no cases of wound infection.	1, 6, 12, 18, and 24 months
Atilgan et al. [29]	Randomized Control Clinical Trial—single blind n = 28 patients treated with PRP injection into the pubocervical fascia and colporraphy—cases n = 28 with colporraphy alone—control group	Anterior repair was performed and 4 mL PRP was injected into the pubocervical fascia.	**POP-Q** system to detect anatomic recurrence, **PFDI** questionnaire for assessment of symptomatic recurrence and **PGI-I** scale for assessment of subjective success. Anatomical success was considered when **point Aa or Ba** was less than −1 according to POP-Q system. **Subjective success** rate was assessed with PGI-I scale. The main outcome was low recurrence rate according POP-Q.	The **Aa and Ba points’** means were significant lower in cases than in controls at 48 months follow-up (*p* = 0.001 and *p* = 0.002, respectively). Symptomatic (**PFDI**) and anatomic recurrence (**POP-Q > 1**) and **reoperation rate** were significantly lower in cases than in controls (*p* = 0.008, *p* = 0.001 and *p* = 0.001, respectively). Only 3.8% of the patients in the cases group had a symptomatic anatomic cystocele recurrence and was reoperated on. Subjective success (**PGI-I)** was significantly higher in cases, with a rate of 89% (*p* = 0.012).	1, 6 months and then annually for 48 months No adverse events were observed.

**Table 2:** Characteristics and data extracted of included studies about PRP in SUI treatment.

Author	Study Type and Patients (n)	Protocol	Scores	Results	Follow-Up from the Baseline (First Injection) and Adverse Events
Grigoriadis T. et al. [32]	Randomized Control Clinical Trial—Double blind n = 25 patients treated with PRP—cases n = 25 with sham (sodium chloride 0.9%)—control group	2 injection sessions—at baseline and 4–6 weeks later. The injections were performed peri-urethrally at the 4-, 6-, and 8-o’clock positions in 3 different levels of the urethra 1–2 cm apart (distal, mid, and proximal).	At the baseline, 3 and 6 months follow-up: **1 h pad test, ICIQ-FLUTS, and KHQ** questionnaire. At 3 and 6 months: **PGI-I** Primary outcome was the subjective evaluation of reported SUI symptoms, indicated by **question 11a of the ICIQ-FLUTS** questionnaire. The level of discomfort during the PRP injections: 10 cm **VAS score.**	**11a question of the ICIQ-FLUTS, incontinence and total ICIQ-FLUTS mean score, PGI-I and 1 h pad test** were significantly improved at 3 months and 6 months in cases and not in the controls (*p* < 0.001). At 6 months follow-up, the **subjective cure rate** was significantly higher in the PRP group (32%) compared to the sham group (4%) (*p* < 0.01). No patient achieved objective cure during the 6-month follow-up, as defined by urine leakage of less than 1 g during the 1 h pad test. Filling and voiding symptom scores (**ICIQ-FLUTS**) did not show significant changes over the follow-up period in either treatment group. No statistically significant differences were observed between the groups in any domain of the King’s Health Questionnaire (**KHQ**).	3 and 6 months No adverse effects were noted after completion of the study
Ashton L. et al. [37]	Randomized Control Clinical Trial—single blind n = 25 patients treated with PRP—cases n = 25 with sham (sodium chloride 0.9%)—control group	Injections were administered into the anterior vaginal mucosa surrounding the mid-urethral region, approximately 1 cm inferior to the urethral meatus, at a depth of approximately 1.5 cm. A total of 2 mL was injected beneath the mid-urethral area, with an additional 1.5 mL administered on each side of the urethra.	**FSFI, the I-QOL survey**, and the **QUID survey**. **VAS for pain/discomfort** for the patients and **VAS for injector difficulty** for the injector. Primary outcomes were as follows: Subjective improvement on the **PGI-I.** A negative **CST** at a bladder volume of 300 mL Secondary outcomes: improvements in the **FSFI scores, I-QOL scores, QUID, VAS for pain, VAS for injector difficulty**.	There was no statistically significant difference in the primary outcomes (**PGI-I improvement and CST**). **FSFI, I-QOL, and QUID** showed no differences between the 2 groups at any of the follow-up Intervals. The average **VAS** for patients and injector for placebo and treatment groups were similar.	1, 3, and 6 months The most common adverse events were vaginal spotting and vaginal discomfort. There was no difference between the placebo and treatment groups
Saraluck A. et al. [34]	Randomized Phase II Clinical Trial—single blind—blinded investigator n = 31 cases treated with PRP + PFMT n = 29 controls treated with PFMT alone	2 injection sessions—at baseline and 4 weeks later. Into the anterior vaginal mucosa surrounding the mid-urethral region, approximately 1 cm inferior to the urethral meatus, at a depth of approximately 1.5 cm. A total of 2 mL was injected beneath the mid-urethral area, with an additional 1.5 mL administered on each side of the urethra. All participants were instructed to perform pelvic floor muscle training (PFMT) three times daily—morning, noon, and evening—completing 8 to 12 contractions and relaxations per session, each lasting 4 to 6 s.	Primary outcome: **objective 1 h pad test** at the baseline and at 5 months follow-up. Secondary subjective outcomes: **I**-**QoL questionnaire score, ICIQ**-**FLUTS question 11a and 11b, PGI**-**I**, and the **percentage subjective improvement score.**	**1 h pad test** after 5 months was **statistically significant** (*p* < 0.05). **I**-**QoL scores** and **11a and 11b in the ICIQ FLUTS** were statistically significantly better in the cases group (*p* < 0.001) and the same was reported for **PGI**-**I scores** (*p* < 0.05). **Self**-**reported percentage of subjective improvement** indicated that 90% of patients in the cases group experienced an improvement in SUI of at least 50%.	5 months No adverse effects were noted after completion of the study.
Athanasiou et al. [33]	Prospective observational pilot study n = 20	2 injection sessions—at baseline (T0) and 4–6 weeks later. The injections were performed into the lower one third of the anterior vaginal wall at 10, 12, and 2 o’clock in 3 different levels of the urethra 1–2 cm apart (distal, mid, and proximal)	At baseline: urodynamic studies, a ICS-standardized **1 h pad test**, and **ICIQ-FLUTS and KHQ questionnaire**. Primary outcome: **question 11a of the ICIQ-FLUTS questionnaire.** At follow-up visits: **1 h pad test, ICIQ-FLUTS and KHQ, and PGI-I** Scale of Improvement. The level of discomfort during the PRP injections as recorded by the 10-cm **VAS score**.	The **11a question score, filling, incontinence, and total mean score of the ICIQ-FLUTS** questionnaire decreased significantly at 3 and 6 months follow-up (*p* < 0.001). **Subjective cure rate** (11a questione ICIQ-FLUTS) **was 10%**, whereas **80%** of the patients **subjectively improved**. The **voiding symptoms score of the ICIQ-FLUTS** did not change significantly throughout the follow-up period. The **PGI-I** showed a significant improvement in 80% of the patients throughout the follow-up period. A mean reduction of 50.2% in urine loss was observed in the **1 h pad test** at the 6 months follow-up and 10% of participants were considered **objectively** cured (defined as urine leakage of less than 1 g during the 1 h pad test). No statistical significance was observed for **KHQ domain,** with the exception of incontinence impact domain.	6 months No adverse effects were noted after completion of the study
Chiang et al. [41]	Prospective observational study n = 26	Every 1 month for a total of 4 treatments within 3 months. One ml of PRP was injected in the urethral sphincter at 5 sites around the urethral meatus (2, 5, 7, 10, and 12 o’ clock positions).	Primary treatment outcomes: **GRA score** (categorized from −3 to +3). GRA score of ≥2 was considered a success. Secondary endpoints: **Subjective parameters**: the **dry rate** after PRP treatments, changes in **SUI VAS, UDI-6 and IIQ-7** from baseline to 3 months after the fourth PRP treatment. **SUI VAS** followed up at 12 months after the fourth PRP treatment. **Objective urodynamic parameters (VUDS and** **ALPP)** at 3 months after the fourth PRP injection (6 months from the baseline)**.**	The **GRA** mean after treatment was 1.5. After 4 PRP 80.8% reported a positive response in alleviating SUI, including 50% that achieved a GRA ≥ 2. **Dry rate**, 46.2% at 3 months and 26.9% at 15 months achieved complete continence and pad-free. 53.8% remained mild SUI with acceptable outcome. **VAS of SUI** was significantly better immediately after the first PRP injection and consolidated by the following repeated injections. The therapeutic effect persisted throughout the 1-year period (*p* < 0.001). At 3 months follow-up, **UDI-6 and IIQ-7** significantly decreased (*p* < 0.001). **VUDS parameters** showed no significant difference between baseline and after PRP treatment. Only **ALPP** was significantly increased (*p* = 0.045).	1, 2, 3, 4, 6, 9, 12, and 15 months Adverse events: only 3.8% reported straining of urination after PRP injection, that resolved after intermittent self-catheterization for several days.
Behnia-Willson et al. [39]	Prospective observational pilot study n = 62 at the baseline and at 3 months follow-up. 37 patients at 1224 months follow-up.	Patients received three treatments, 4 to 6 weeks apart (within 2–3 months) with a 90° vaginal laser-emitting probe inserted up to the level of the bladder neck, rotated and withdrawn, exposing the anterior lower one third of the vagina to the laser. Participants were also subjected to total vaginal length laser treatments with a 360° probe. Participants also received the same amount of PRP immediately after each vaginal laser treatment. PRP was injected into the anterior lower one third of the vagina and peri-urethral area.	**APFQ Questionnaire:** at baseline, 3 months after the third treatment (5–6 months from the baseline), and at a 12–24 months after treatment. Primary treatment outcome: **question 6 of the APFQ**, which indicates Stress Incontinence. The secondary treatment outcomes: **relevant items of the APFQ.**	**Question 6 APFQ**: At the baseline, 0% reported either occasional or no stress urinary symptoms and 100% reported frequently or daily symptoms. At 3 months, 66.2% reported occasional or no symptoms. At 12–24 months, 62.1% of the patients report either occasional or no symptoms. The improvement was statistically significant (*p* < 0.001) All the symptoms (**APFQ**) improved significantly (*p* < 0.001) within the baseline and at 3 months follow-up and then from 3 to 12–24 months, except for pad usage, which did not improve from 3 to 12–24 follow-up (*p* = 0.073).	5/6 and 12/24 months No adverse effects were noted after completion of the study.
Long CY et al. [35]	Prospective interventional pilot study n = 20	Injections were administered into the anterior vaginal mucosa surrounding the mid-urethral region, approximately 1 cm inferior to the urethral meatus, at a depth of approximately 1.5 cm. A total of 2 mL was injected beneath the mid-urethral area, with an additional 1.5 mL administered on each side of the urethra.	Primary outcome: **ICIQ-SF, UDI-6, IIQ-7, OABSS, and POPDI-6**. Only 40% of the women completed the **urodynmaic studies** before and 6 months after intervention. Secondary outcome: sexual function investigated before and after the treatment by **FSFI** questionnaire.	**ICIQ-SF, UDI-6, and IIQ-7** showed significant incontinence improvement at both 1 month and 6 months post-treatment. **OABSS** had an improvement only at 1 month follow-up. **POPDI-6** did not show statistically significant improvement. **ICIQ-SF** particularly demonstrated an efficacy of 60% in symptoms improvement. In the **urodynamic studies**, residual urine and bladder volume at first sensation to void increased significantly. All other urodynamic parameters showed no significant differences following treatment. No significant changes were reported in the **FSFI questionnaire**.	1 and 6 months No adverse effects were noted after completion of the study.
Ural et al. [36]	Prospective interventional study n = 34	i-PRF was injected 3 times, separated by 1 month, into the anterior vaginal mucosa under the mid-urethral, 1–2 cm below the urethra meatus. Half of the dose was administered under the mid-urethra, and the remaining quarter was administered into the right and left sides of the mid-urethra.	**ICIQ-SF, UDI-6, IIQ-7, and POPDI-6** questionnaires. Primary outcome: **ICIQ-SF**. If a woman felt no symptoms of SUI after surgery, she was considered cured. Secondary outcomes included **UDI-6, IIQ-7,** and **POPDI-6.** The results of the study were not evaluated with clinical parameters such as urodynamics and pad test.	According to **ICIQ-SF,** the rate of clinical improvement was 79.4% at 1 month and 64.7% at 6 months after treatment. **ICIQ-SF, UDI-6, IIQ-7,** and **POPDI-6** scores at 1 and 6 months post-treatment were significantly lower than pre-treatment values (*p* < 0.001). No significant changes were observed in the scores between 1 and 6 months following treatment.	1 and 6 months No adverse effects were noted after completion of the study.
Daneshpajooh et al. [40]	Prospective randomized controlled clinical trial. n = 10 patients received PRP—case group n = 10 patients received mid-urethral sling procedure—control group.	3 mL of PRP was injected via cystourethroscopy at four sites along the mid-urethra using endoscopic needles. Three patients in the treatment group consented to receive a subsequent dose. The second injection was administered one month after the initial injection, and in one case, a third injection was performed one month after the second.	**ICIQ, I-QOL,** **UDI-6,** and **cough stress test.**	In PRP group **cough stress test** at 1 month, results negative for 70% of the patients. In the control group, the results were negative for 80%. The results were not significantly different between 1 month and 3 months follow-up. **ICIQ, I-QoL, and UDI-6** results were significantly different before and after treatments in both groups. However, the response to the mid-urethral sling procedure was better and the difference was statistically significant.	1 and 3 months
Tahoon A.S. et al. [38]	Prospective observational study n = 26	One injection was administered into the anterior vaginal wall, near the external urethral sphincter, at three sites on each side. An additional injection was performed in the paraurethral area, with a depth of 10 mm from lateral to the external urethral orifice.	**Urodynamics study** changes before and three months after treatment. **1 h pad test** before and after PRP injection. **ICIQ-SF, UDI-6 IIQ-7, OBSS, and FSFI** after 1 and 3 months.	50% of the women completed the urodynamics studies before and 3 months after intervention. **Residual urine** and **bladder volume at first sensation to void** increased significantly. All other urodynamics parameters showed no significant differences. After the PRP treatment, the average **1 h pad test** was 2.55 g (−2.8 g compared to initial value). **ICIQ-SF, UDI-6 IIQ-7, OBSS, and FSFI** showed significant incontinence improvement at both 1 and 3 months. According to **ICIQ-SF**, 30% were cured, 25% reported mild symptoms, 40% reported moderate and 5% severe diseases. Improvement occurred in 54% patients and no change in 46%. No significant changes before and after the treatment were reported by **FSFI**	1 and 3 months No adverse effects were noted after completion of the study.

## Supplementary Materials

The following supporting information can be downloaded at: https://www.mdpi.com/article/10.3390/jpm15060214/s1, Table S1: PRISMA-ScR; Table S2: JBI Critical Appraisal Checklist; Table S3: Demographic table-POP; Table S4: Demographic table-SUI.

## Abbreviations

The following abbreviations are used in this manuscript:

POP	Pelvic Organ Prolapse
SUI	Stress Urinary Incontinence
PRP	Platelet-rich plasma
RCT	Randomized Controlled Trial
FDA	Food and Drug Administration
ICS	International Continence Society
UI	Urinary Incontinence
PFMT	Pelvic Floor Muscle Training
BMI	Body Mass Index
POP-Q	Pelvic Organ Prolapse Quantification
APG	Autologous Platelet Gel
PGI-I	Patient’s Global Impression of Improvement
VAS	Visual Analogue Scale
P-QoL	Prolapse Quality of Life
ICIQ-FLUTS	Incontinence Questionnaire—Female Lower Urinary Tract Symptoms
KHQ	King’s Health Questionnaire
CST	Cough Stress Test
FSFI	Female Sexual Function Index
QUID	Questionnaire for Urinary Incontinence Diagnosis
I-QoL	Incontinence Quality of Life
PFMT	Pelvic Floor Muscle Training
UDI-6	Urinary Distress Inventory
GRA	Global Response Assessment
IIQ-7	Incontinence Impact Questionnaire
VUDS	Videourodynamic Study
ALPP	Abdominal Leak Point Pressure
APFQ	Australian Pelvic Floor Questionnaire
ICIQ-SF	Incontinence Questionnaire-Short Form
OABSS	Overactive Bladder Symptom Scores
POPDI-6	Pelvic Organ Prolapse Distress Inventory 6
FSFI	Female Sexual Function Index
