# Supplementary material for: PRP Therapy for Stress Urinary Incontinence and Pelvic Organ Prolapse: A New Frontier in Personalized Treatment?
Source: J Pers Med. 2025 May 22;15(6):214. doi: 10.3390/jpm15060214 (PMC12194431; doi:10.3390/jpm15060214)
Supplement: Supplementary file 1 [file jpm-15-00214-s001.zip › Demographic POP - Table S3.pdf]

Author	Demographic Characteristics of the Participants	Inclusion and Exlusion Criteria	Kit used
Einarsson J.I. et al 2009	Age, mean 48,6<br><br>BMI, mean 25,2<br><br>Parity, mean 2,4<br><br>Menopause 66,7%<br><br>Concomitant surgery (posterior repair, vaginal hysterectomy, or vaginal enterocele repair), 77,8%	Cystocele in patients younger than 55 years; who had not undergone previous corrective surgery for cystocele.<br><br>Exclusion criteria: younger than 18 years and older than 55 years; undergoing emergency surgery; known intravenous drug users.	NS / Thrombin-rich serum and platelet-rich plasma were mixed to form (Autologous Platelet Gel) APG.<br><br>Collagen content as micrograms of hydroxyproline was determined from hydroxyproline (Sigma) standard curve.
Gorlero F. Et al. 2012	Age, mean 62.7<br><br>BMI, mean 27<br><br>Parity, mean 1.8<br><br>Menopause 100%, for a mean of 13.3 years<br><br>Stage III POP-Q 60%<br><br>Stage II POP-Q 40%<br><br>Concomitant Surgery (anterior repair, enterocele repair, apical repair, perineal body repair, reductive plastic surgery, SUI surgery, posterior repair or transverse perineal muscle plication), 100%	POP-Q stage II or higher in patients with previous prolapse surgery and high risk for: recurrence, erosion with graft materials, intraoperative and postoperative complications with traditional pelvic reconstructive surgical procedures (anesthetic and bleeding risks).<br><br>All patients had previous vaginal hysterectomy and concomitant pelvic reconstructive surgery for prolapse without any graft materials.	Vivostat system (Vivostat A/S, Denmark) for preparation and application of PRF (6ml of autologous sealant).<br><br>The PRF was sprayed directly onto the surgical site, where it polymerized on contact.
Atilgan et al. 2020	Age, mean 58.2<br><br>BMI, mean 25.6<br><br>Parity, mean 3.5<br><br>Stage of cystocele, median 2 (range 1-4)<br><br>Menopause 67%<br><br>Preoperative POP-Q stage, point Aa +3<br><br>Preoperative POP-Q stage, point Ba +3.6	Cystocele repair.<br><br>Exlusion criteria: Cystocele repair performed for other prolapse compartments and concomitant stress urinary incontinence surgery or history of POP surgery.	Vacutainer Kit (10% of calcium chloride, 0.2 ml, was added to 4 ml of PRP to activate platelets).
