# Supplementary material for: PRP Therapy for Stress Urinary Incontinence and Pelvic Organ Prolapse: A New Frontier in Personalized Treatment?
Source: J Pers Med. 2025 May 22;15(6):214. doi: 10.3390/jpm15060214 (PMC12194431; doi:10.3390/jpm15060214)
Supplement: Supplementary file 1 [file jpm-15-00214-s001.zip › Demographic SUI - Table S4 .pdf]

Author	Demographic Characteristics of the Participants	Inclusion and Exlusion Criteria	Kit used
Grigoriadis T. et al 2024	Age, mean 55,4<br>BMI, mean 26,5<br>Parity, mean 1,92<br>Menopause 72%<br>VAS, mean, during PRP injection 2,73	SUI - in women presenting with pure or predominant SUI, without pelvic organ prolapse (<2 POPQ), genitourinary fistula, anti-incontinence surgery, detrusor overactivity on urodynamics, previous anti-incontinence and prolapse surgery (including hysterectomy).<br>Exlusion criteria were severe bleeding disorders, active malignant pathology, pregnancy, treatment with hormone therapy ot anticoagulant treatment that could't be discontinue for 5–7 days.	OMNIPLEX Gyno kit for PRP preparation (3 mL of PRP is produced with a platelet concentration 9 times higher than baseline platelet count)
Athanasiou et al. 2021	Age, mean 56.5<br>Weight, mean, kg 71.5<br>BMI, mean 27,1<br>Parity, mean 2.05<br>VAS, mean, during PRP injection 2,73	SUI - in adult, nonpregnant women with a history of failed conservative treatment.<br>Exclusion criteria: active malignant pathology, undiagnosed abnormal uterine bleeding, genitourinary fistula, anti-incontinence surgery, use of antiplatelet or anticoagulant medication, nonsteroidal anti-inflammatory drugs, pelvic organ prolapse stage >2 according to the Pelvic Organ and detrusor overactivity on urodynamics..	RegenKit BCT-3 for PRP preparation (5 mL of PRP is produced and activated by adding a 1:10 volume of calcium chloride 10%.)
Saraluck A. Et al 2023	Age, mean 51,76<br>BMI, mean 26,83<br>Parity, 75% had at least one vaginal delivery<br>Previous hysterectomy 10% of the patients<br>Menopause 65% of the patients<br>Brink score, mean 8.79<br>VAS, mean, during PRP injection 2,76	SUI - in patients with mild to moderate SUI, a 1-h PWT of >1 g but not >50 g.<br>Exlusion criteria: history of treatment with A-PRP or any other method for SUI, any suspicion of urgency symptoms or urge urinary incontinence, Overactive Bladder Symptoms or pelvic organ prolapse, heavy continuous urine leakage, obesity, thrombocytopenia, or coagulopathy, anemia, urinary tract infection, undiagnosed abnormal vaginal bleeding and treatment with corticosteroids or immunosuppressants.	RegenKit BCT-3 for PRP preparation (5 mL are produced with a platelet concentration of ×1.6).
Chiang et al. 2022	Age, mean 61,7	SUI - in patients with ISD (intrinsic splicer deficiency) with urinary leakage in association with urethral incompetence without bladder hypermobility during abdominal straining, refractory to conservative treatment or that had SUI recurrence after previous surgical procedure by suburethal sling at least 1 year before the study and patient with predominant stress urinary incontinence for more than one year.<br>Patient with severe POP, critical thrombocytopeni, anticoagulant use or hypofibrinogenemia, active urinary tract infection or any contraindication for urethral catheterization during the study were excluded.	Not specified / 50 ml of peripheral blood was withdrawn, followed by two centrifugation steps (5 mL are produced with a platelet concentration of x 2.5–5).
Behnia-Willson et al. 2019	Age, mean 55,98<br>Postmenopausal 77.4%<br>Parity, 92% was multiparous<br>BMI, 65,2% was obese<br>Urodynamic assessment at the baseline in 56,5% of the patients	SUI - in patient with clinically significant symptoms of SUI, positive cough test, urethral hypermobility and them who not respond or responded unsatisfactorily to previous therapies.<br>Exclusion criteria: current malignancy, known cervical dysplasia, acute or recurrent urinary tract infections, POPQ stage > I, previous pelvic reconstructive surgery, undiagnosed abnormal uterine bleeding, current pregnancy, concurrent use of anti-platelet or anti-coagulant medications.	Transvaginal laser treatments (MonaLisa Touch, SmartXide2 V2LR, DEKA, Italy).<br>Regen PRP (NS the injection volume)
Long CY et al. 2021	Age, mean 44,5<br>BMI, mean 22,7<br>Parity, mean 1,6<br>Pad test, mean 5,8g<br>Menopause 25% of the patients<br>VAS, mean, during PRP injection 2,73	SUI in patients over 20 years old.<br>Exclusion crriteria: known platelet dysfunction, critical thrombocytopeni, hypofibrinogenemia, altered Prothrombine Time, hemodynamic instability, sepsis, acute or chronic infections, chronic liver disease, anti-coagulant users and known malignancy.	RegenKit for PRP preparation (5 mL are produced with a platelet concentration of ×1.6).
Ashton L. et al. 2024	Age, median 48<br>BMI, mean 29<br>Previous Hysterectomy 12%<br>VAS, mean, during PRP injection 2,25	SUI - patient in patient with predominant SUI at a bladder volume less than 300 mL and post-void residual less than 150 mL.<br>Excluding criteria: no urinary infection and pregnancy.	Arthrex Autologous Conditioned Plasma Double Syringe System (5 mL of the subject's PRP was separated at the end of the process).
Ural et al. 2024	Age, mean 51.5<br>BMI, mean 30.25<br>Parity, mean 3.0<br>Menopause 50%	SUI in patients with a positive cough-stress test, had previously conservative treatment and had been exhibiting SUI for longer than six months.<br>Exclusion criteria: history of platelet dysfunction, thrombocytopenia, hypofibrinogenemia, acute or chronic infections, chronic liver disease, anticoagulant or antiplatelet use, malignancy, neurogenic urinary incontinence, a history of previous surgery for SUI and pelvic organ prolapse.	NS/The blood was centrifuged for 3 min at 700 rpm (60 × g force) at room temperature using Intra-Spin by Intra-Lock International in Boca Raton, Florida, USA.<br>4ml of i-PRF were extracted from each tube.
Daneshpajooh et al. 2021	Age, mean 50.9<br>Parity, mean 4.4	SUI - in patient with primary symptoms of SUI confirmed by patient's medical history and clinical symptoms, including a focused incontinence evaluation; age range of 30-65 years; and patient's willingness to undergo surgical treatment.<br>The exclusion criteria were: residual urine volume exceeding 100 cc after excretion; evidence of detrusor over-activity on the urodynamic test; history of suburethral sling procedure or other urogenital surgeries; active urinary tract or vaginal infection; malignancies of the genitourinary system; history of hemorrhagic disorders; recent history of genitourinary fistula or urethral diverticulum; advanced pelvic organ prolapse on the pelvic examination; uncontrolled diabetes mellitus, and any other contraindications for surgical procedures.	NS/The blood was centrifuged 2 times and 3 ml of PRP was preparate.
Tahoon A.S. et al. 2022	Age, Mean 48.65<br>Parity, Mean 4.65<br>BMI, Mean 29.4<br>Pad test, mean 5.35g<br>Menopause 50%	SUI - in patient with mild to moderate SUI above 35 years old<br>Exclusion criteria: anti-platelet agent treatment, platelet dysfunction syndrome, critical thrombocytopenia, acute and chronic infections, collagen disease, anti-coagulation therapy and history of malignancy	Golden VAC for PRP preparation (4 mL)
