# Supplementary material for: PRP Therapy for Stress Urinary Incontinence and Pelvic Organ Prolapse: A New Frontier in Personalized Treatment?
Source: J Pers Med. 2025 May 22;15(6):214. doi: 10.3390/jpm15060214 (PMC12194431; doi:10.3390/jpm15060214)
Supplement: Supplementary file 1 [file jpm-15-00214-s001.zip › JBI PRP Table S2 .pdf]

Title	Author, Year	D1	D2	D3	D4	D5	D6	D7	D8
Platelet-Rich Plasma for the Treatment of Stress Urinary Incontinence—A Randomized Trial	Grigoriadis T. et al 2024	YES	YES	YES	YES	YES	YES	YES	YES
The Use of Platelet-rich Plasma as a Novel Nonsurgical Treatment of the Female Stress Urinary Incontinence: A Prospective Pilot Study	Athanasiou et al. 2021	YES	YES	YES	YES	YES	YES	YES	YES
Autologous platelet rich plasma (A-PRP) combined with pelvic floor muscle training for the treatment of female stress urinary incontinence (SUI): A randomized control clinical trial	Saraluck A. Et al 2023	YES	YES	YES	YES	YES	YES	YES	YES
The Efficacy and Mid-term Durability of Urethral Sphincter Injections of Platelet-Rich Plasma in Treatment of Female Stress Urinary Incontinence	Chiang et al. 2022	UNCREAR	YES	YES	YES	UNCLEAR	YES	YES	YES
Promising impact of platelet rich plasma and carbon dioxide laser for stress urinary incontinence	Behnia-Willson et al. 2019	YES	YES	YES	UNCLEAR	UNCLEAR	YES	YES	YES
A pilot study: effectiveness of local injection of autologous platelet-rich plasma in treating women with stress urinary incontinence	Long CY et al. 2021	YES	YES	YES	YES	YES	YES	YES	YES
A Single Injection of Platelet-rich Plasma Injection for the Treatment of Stress Urinary Incontinence in Females: A Randomized Placebo-controlled Trial	Ashton L. et al. 2024	YES	UNCLEAR	YES	YES	YES	YES	YES	YES
The effect of injectable platelet rich fibrin as a nonsurgical treatment of the female stress urinary incontinence	Ural et al. 2024	YES	YES	YES	YES	UNCLEAR	YES	YES	YES
The Effect of Periurethral Injection of Pure Platelet-rich Plasma in the Treatment of Urinary Incontinence in Female Patients: a randomized clinical trial	Daneshpajooh et al. 2021	UNCREAR	YES	YES	UNCLEAR	YES	YES	YES	YES
Use of Autologous Platelet Gel in Female Pelvic Organ Prolapse Surgery: A Feasibility Study	Einarsson J.I. et al 2009	YES	YES	YES	YES	UNCLEAR	YES	YES	YES

Title	Author, Year	D1	D2	D3	D4	D5	D6	D7	D8
New approach in vaginal prolapse repair: mini-invasive surgery associated with application of platelet-rich fibrin	Gorlero F. Et al. 2012	YES	YES	YES	YES	YES	YES	YES	YES
Cystocele Repair with Platelet-Rich Plasma	Atilgan et al. 2020	YES	YES	YES	YES	YES	YES	YES	YES
